# Forecasting Climatic Trends Using Neural Networks: An Experimental Study Using Global Historical Data

**DOI:** 10.3389/frobt.2019.00032

**Published:** 2019-04-26

**Authors:** Takeshi Ise, Yurika Oba

**Affiliations:** ^1^Field Science Education and Research Center (FSERC), Kyoto University, Kyoto, Japan; ^2^Japan Science and Technology Agency (JST), Kawaguchi, Japan

**Keywords:** climate change, neural networks, big data, historical data, NVIDIA DIGITS, top-down approach, graphical image classification, global environmental change

## Abstract

Climate change is undoubtedly one of the biggest problems in the 21st century. Currently, however, most research efforts on climate forecasting are based on mechanistic, bottom-up approaches such as physics-based general circulation models and earth system models. In this study, we explore the performance of a phenomenological, top-down model constructed using a neural network and big data of global mean monthly temperature. By generating graphical images using the monthly temperature data of 30 years, the neural network system successfully predicts the rise and fall of temperatures for the next 10 years. Using LeNet for the convolutional neural network, the accuracy of the best global model is found to be 97.0%; we found that if more training images are used, a higher accuracy can be attained. We also found that the color scheme of the graphical images affects the performance of the model. Moreover, the prediction accuracy differs among climatic zones and temporal ranges. This study illustrated that the performance of the top-down approach is notably high in comparison to the conventional bottom-up approach for decadal-scale forecasting. We suggest using artificial intelligence-based forecasting methods along with conventional physics-based models because these two approaches can work together in a complementary manner.

## Introduction

Because climate change is the biggest environmental problem currently, it has attracted interest from many researchers and policymakers. For climate forecast, physics-based models have been widely used. General circulation models (GCMs) have been constructed by numerical representations of atmospheric physical conditions (Manabe et al., 1965). Earth system models (ESMs) are advanced models based on GCMs and are mainly used for current climatic studies (e.g., Collins et al., 2006), which considers features such as biogeochemical cycling and atmospheric chemistry. These models are based on the laws of physics such as conservation of mass, energy, and momentum. These models can be referred to as bottom-up approaches because they forecast climate using physical boundary conditions. Although the performance of ESMs is improving, these models still suffer from significant forecast uncertainties. Such uncertainties in future climate may delay amelioration and adaptation to climate change (Intergovernmental Panel on Climate Change, 2013).

There should be another approach for climate forecasting; a top-down, phenomenological approach can complement a bottom-up, mechanistic approach. The most intuitive top-down approach is using statistical models such as regression analysis. For forecasts, there are many statistical approaches for time series, such as Autoregressive Integrated Moving Average. However, a top-down approach is not mainstream for the current research as its forecast ability is believed to be limited, especially under novel environmental conditions. Still, there are several interesting examples of the top-down approach. Sévellec and Drijfhout (2018) predicted climatic trends by probabilistic forecast where trained researchers “picked” seemingly important trends and expressed them in a statistical manner.

We believe that using deep neural networks (DNNs) can be an excellent tool for the top-down approach because DNNs are proven to be very successful in artificial intelligence (LeCun et al., 2015). For example, deep learning has revolutionized computer vision (e.g., Ise et al., 2018), chemical engineering (e.g., Pławiak and Rzecki, 2015), and medical diagnosis (e.g., Yıldırum et al., 2018). It has attracted many users as it has a variety of libraries and computational environments, especially for image detection and classification. DNNs have been used for time-series analysis (e.g., Sak et al., 2014). However, these approaches are mostly unsuitable for finding large-scale features in climatic trends. Although there are a few studies where machine learning has been utilized in parametrization of atmospheric models (Gentine et al., 2018; Rasp et al., 2018), DNNs are not fully utilized in climate studies currently.

In this study, we apply a simple but novel approach to determine climatic trends. Using global historical data of mean monthly temperature for the years 1901–2016, we graphically represent the temperature dynamics and feed these images to a DNN. We classify the images in two categories: when a temperature rise is observed after the training period and when a temperature fall is observed after the training period. This study is a pedagogical experiment as we know the “correct” answer; we verify whether the DNN successfully determines the answer. We test whether this top-down approach can be a tool for phenomenological forecast of future climate. We also test the forecast performance in limited spatiotemporal scales to find specific timings and locations for which the performance is unusually good or bad. Our approach is unique because it employs a DNN-based, top-down approach for climate forecasts. Our aim is to illustrate the performance and characteristics of this new approach and to contribute to the studies on climate change.

## Methods

**Figure 1:**
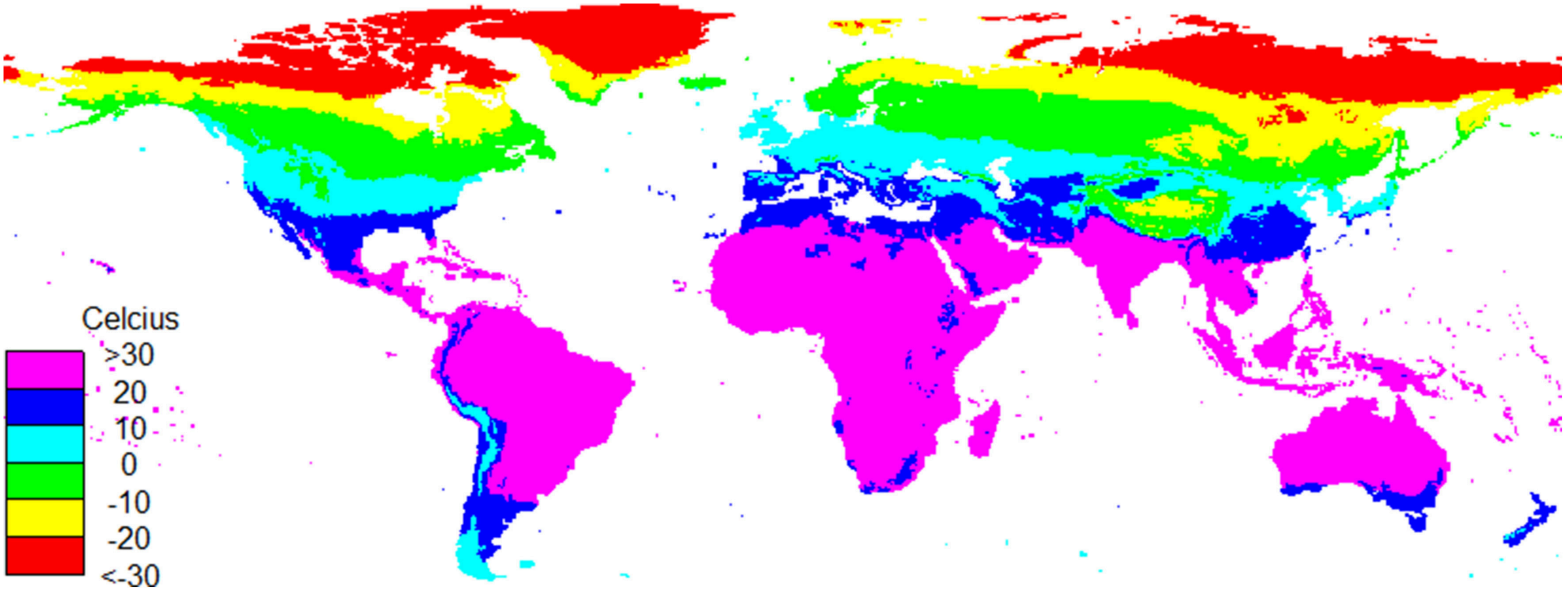
Global mean monthly temperature in March 2001.

**Figure 2:**
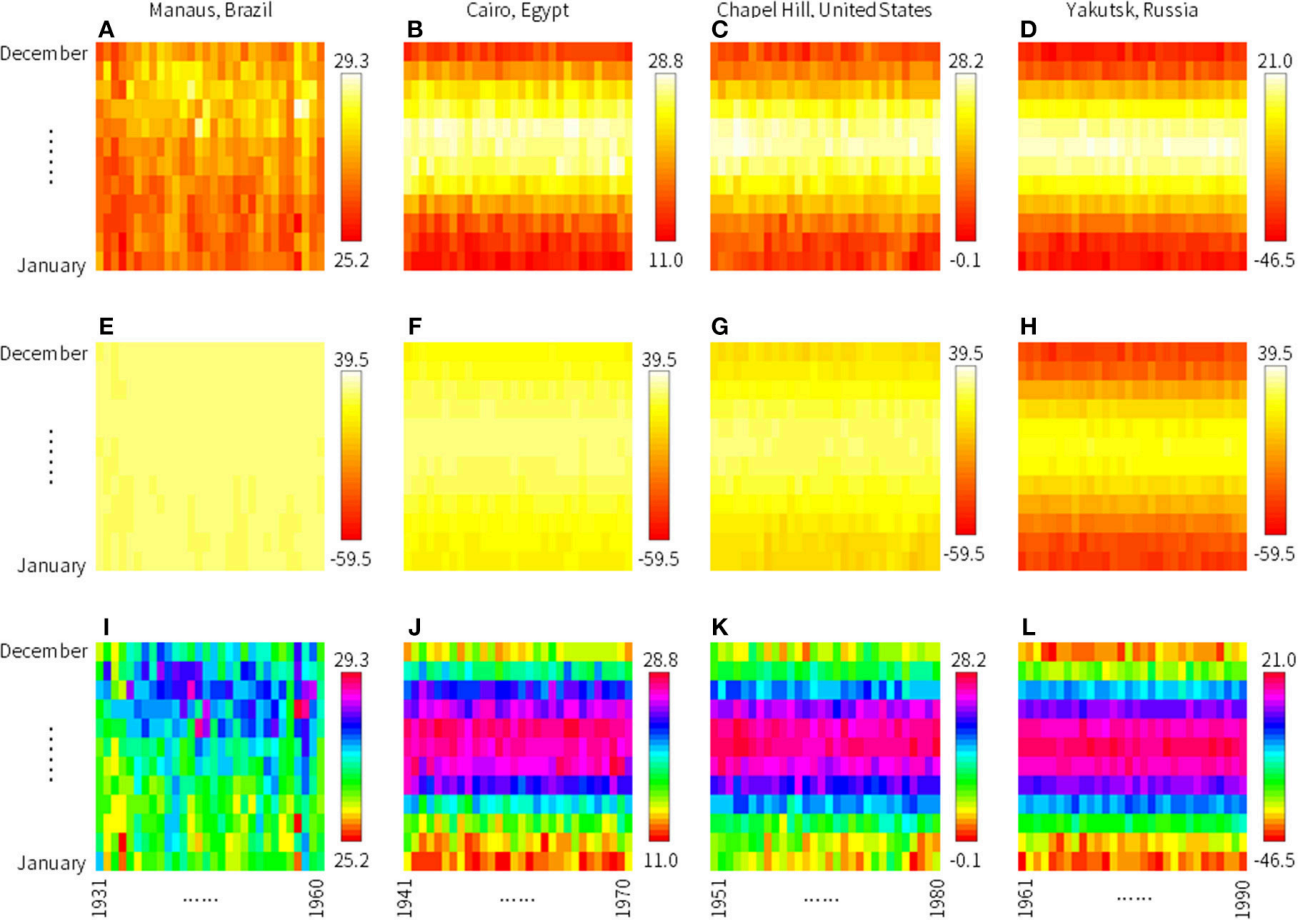
Some examples of training images. To illustrate the characteristics of climate zones, we show images of 30-year mean monthly temperature of grids including **(A,E,I)** Manaus, Brazil, **(B,F,J)** Cairo, Egypt, **(C,G,K)** Chapel Hill, United States, and **(D,H,L)** Yakutsk, Russia to represent tropical rain forest, desert, moist temperate, and boreal, respectively. For **(A–D)**, the upper and lower limits of the color scheme are set automatically by software R. These images may look similar but note the difference in temperature ranges shown in the legends. For **(E–H)**, the upper and lower limits of the color scheme are set manually to the universal maximum and minimum temperatures of the entire dataset. For **(I–L)**, the color scheme rainbow is used.

The input data used for this experiment was the global mean monthly temperature data (CRU TS 4.01) from the Climatic Research Unit (Harris et al., 2014). This dataset covers the global terrestrial area in 0.5° × 0.5° grids (Figure 1). Using this dataset, we randomly selected a place and start time for each graphical representation. In one graphical representation, the mean monthly temperature of the selected place was retrieved, and data for 30 years (training period) from the start time were used for the graphical representation; we created an image of 60 × 60 pixels from the temperature data (Figure 2) using R 3.4.4 (R Core Team, 2018). Then, we classified the images into two categories (RISE and FALL) based on the mean temperature for 10 years after the training period. The assigned categories were used as training data paired with the training images for the DNN. The hardware to run and test the DNN had XEON E5-2630v3 CPU, 16 GB RAM, and NVIDIA Quadro K620 GPU; the operating system was Ubuntu 14.04 LTS. The settings and parameters for NVIDIA DIGITS 6.0 (Caffe version: 0.15.13), the platform for the DNN, are summarized in Table 1. We employed convolutional neural network (CNN) with LeNet (LeCun et al., 1998). Randomly selecting 25% of images for validation, accuracy, and loss are calculated in each training epoch. We set the number of training epoch to 30, with systematically decreasing learning rates.

**Table 1:** NVIDIA DIGITS 6.0 settings and parameters.

% validation images	25
Image encoding	png
DB backend	lmdb
DB compression	none
Training epochs	30
Snapshot interval	1
Validation interval	1
Random seed	none
Solver type	stochastic gradient descent
Base learning rate	0.01
Network	LeNet (LeCun et al., 1998)

*In this framework, there is a choice for the DB backend (data format for the database): lmdb and hdf5. We created the database in the former format. Although we set snapshot interval = 1 to store the model for each training epoch, we only used the models from the last (30th) training epoch for analyses in this study. We set validation interval = 1 to calculate accuracy for each training epoch. We did not set the random seed, and the models for this study were individually initialized. We used the stochastic gradient descent solver (default); other choices were Nesterov's accelerated gradient, adaptive gradient, AdaDelta, adaptive moment estimation, and RMSprop. The base learning rate is the parameter to determine efficiency for learning. We set the parameter to 0.01 (default). The network we used was LeNet (LeCun et al., 1998). Other network choices were AlexNet (Krizhevsky et al., 2012) and GoogLeNet (Szegedy et al., 2014). See Discussion for the comparison*.

There are 67,420 terrestrial grid cells in a resolution of 0.5°. The length of the time series is 116 years. When we systematically shift the window of 40 years (30 years for training images and 10 years to assign categories: RISE and FALL), there can be 116–40 +1 = 77 different training images. Thus, the maximum number of the training images is 67,420 × 77 = 5,191,340. To accelerate the series of experiments in this study, we randomly chose subsets of training images by assigning a random number *c* from 0 to 1 for each training image in each experiment. Only when *c* > *c*_*t*_, where *c*_*t*_ is the threshold, the ith training image was chosen for the subset. For example, when *c*_*t*_ = 0.99, approximately 1% of the training images (~51,913 images) will be selected as the subset. We analyzed the data to determine the effect of *c*_*t*_.

We also performed analysis to see the difference in the color schemes in R (heat.colors, topo.colors, and rainbow). Moreover, we tested the effect of upper and lower limits of the images. In the default condition, upper and lower limits of an image plot are automatically defined by the “image” function in R, according to the monthly temperature data. In this experiment, we created an alternative condition where universal highest and lowest temperatures of the entire dataset were set as the upper and lower limits of all images; global maximum and minimum temperatures for 1901–2016 were 39.5°C and −59.5°C, respectively. The images for Manaus, Brazil (Figures 2A,E,I), for example, were drawn using identical temperature data. Although the differences were only from the plotting scheme (upper and lower limits and color scheme), these differences somewhat affected the resultant classification accuracy.

Moreover, we made classification experiments with space and time restrictions. For spatial limitation, we selected four climatic zones (Amazon as tropical rainforest, Sahara as desert, Eastern US as temperate, and Siberia as boreal) and evaluated the differences in the model performance. For temporal limitation, we restricted the starting year to a single decade (1900s−1960s) and compared the model performance.

## Results

**Figure 3:**
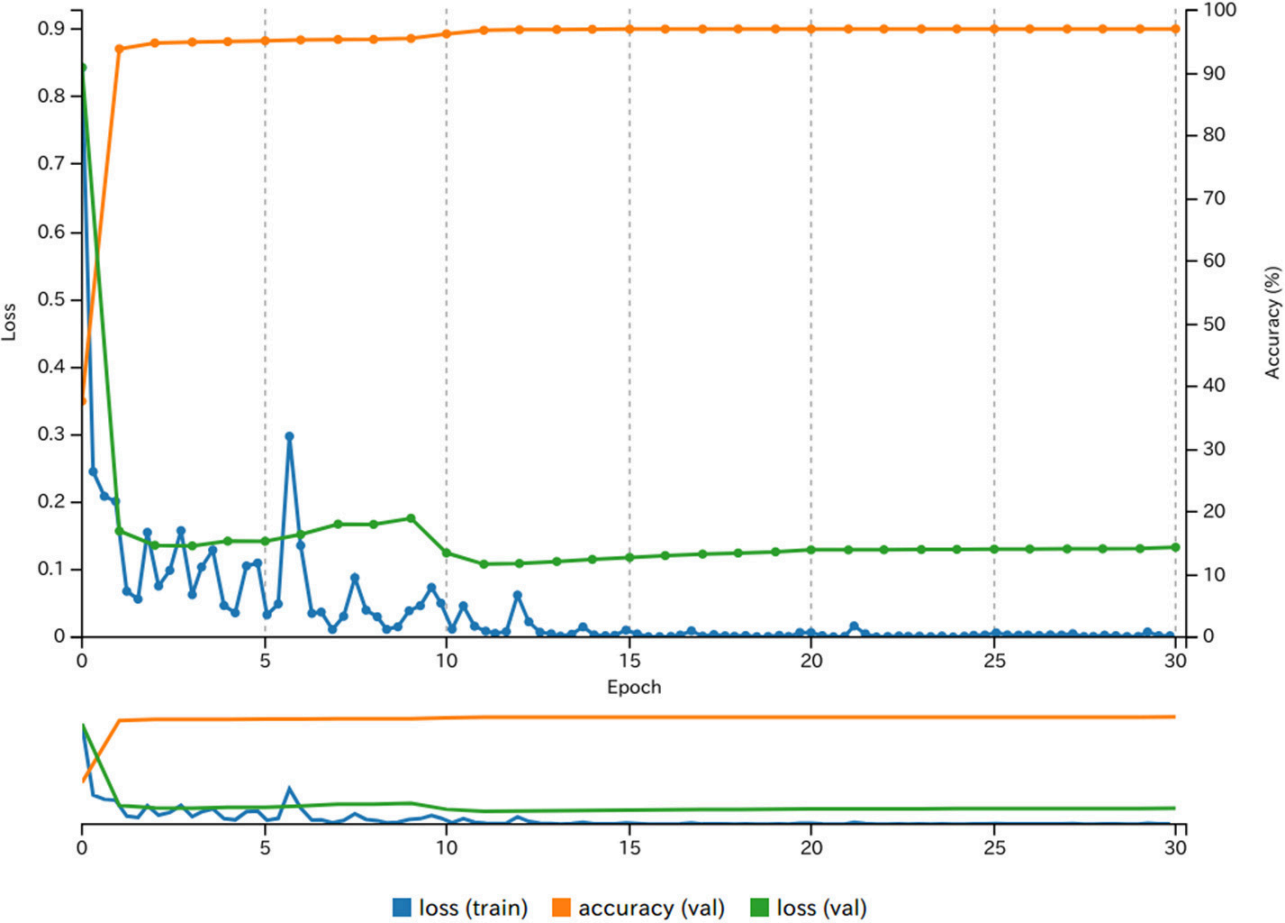
Development of the image classification for the best model (MODEL4).

The training process of the DNN in NVIDIA DIGITS 6.0 was successful in classifying images into RISE and FALL categories. The greater number of training images involved, the higher was the attained accuracy of classification (Table 2). We set MODEL3 as the reference model for the following experiments. With the hardware and software configurations for this study (see Methods and Table 1), the time required to train MODEL3 was 13 min. For MODEL4, the required time was increased to 133 min, showing that the training times were linearly correlated to the number of training images. For training of MODELs 1–24, the same settings such as DNN parameters and structure were used (see Table 1). Figure 3 shows the learning process of DIGITS 6.0 with 30 epochs.

**Table 2:** Summary of classification experiments.

**Model number**	***c_***t***_***	**Spatiotemporal restrictions**	**Number of training images**			**Image design**	**Accuracy (%)**
			**FALL**	**RISE**	**Total**		
MODEL1	0.9999	none	141	328	469	default	65.625
MODEL2	0.999	none	1,524	3,573	5,097	default	78.984
MODEL3	0.99	none	15,685	36,206	51,891	default	92.026
MODEL4	0.9	none	157,042	362,676	519,718	default	97.037
MODEL5	0.9999	none	148	342	490	topo.colors	72.656
MODEL6	0.999	none	1,581	3,588	5,169	topo.colors	77.210
MODEL7	0.99	none	15,525	36,414	51,939	topo.colors	91.441
MODEL8	0.9	none	157,154	362,716	519,870	topo.colors	96.762
MODEL9	0.9999	none	146	325	471	rainbow	70.313
MODEL10	0.999	none	1,598	3,529	5,127	rainbow	79.040
MODEL11	0.99	none	15,593	36,378	51,971	rainbow	91.818
MODEL12	0.9	none	157,028	361,762	518,790	rainbow	96.913
MODEL13	0.99	none	15,623	36,026	51,649	fixed universal min and max	88.165
MODEL14	0.3	Amazon	15,020	36,851	51,871	default	98.468
MODEL15	0.907	Sahara	17,259	34,724	51,983	default	97.551
MODEL16	0.1	Eastern US	21,887	29,935	51,822	default	97.145
MODEL17	0.946	Siberia	13,799	37,999	51,798	default	93.366
MODEL18	0.923	1900s	8,414	43,605	52,019	default	96.821
MODEL19	0.923	1910s	22,810	29,481	52,291	default	94.988
MODEL20	0.923	1920s	30,693	21,400	52,093	default	95.393
MODEL21	0.923	1930s	35,492	16,161	51,653	default	95.862
MODEL22	0.923	1940s	16,961	34,898	51,859	default	95.797
MODEL23	0.923	1950s	4,801	46,813	51,614	default	98.059
MODEL24	0.923	1960s	1,045	50,724	51,769	default	98.974

*For image design, the color scheme “default” is for heat.colors, and the maximum and minimum temperatures are set automatically. MODEL3 is the reference model and MODEL4 is the best model. MODEL13–MODEL24 are compared with MODEL3 because these models use approximately the same number of training images*.

The performance of the models improved with the increase in the number of training images. However, the change in accuracy per unit number of training images gradually decreased, suggesting saturation. This suggests that researchers should consider cost performance because accuracy and computational burden have a tradeoff relationship.

We tested the effect of color schemes in R. Overall, heat.colors showed the best performance among other color schemes. This implies that the color scheme of artificially generated images can affect the performance of DNNs. We also noted that topo.colors was not the best color scheme when the number of training images was small.

Next, using the reference model (MODEL3) settings, we limited the time or space of the training data to determine the performance in specific conditions. We focused on specific climate zones (MODEL14–MODEL17): tropical rain forest (Amazon, 0-12S, 50-70W), desert (Sahara 12-26N, 10W-30E), temperate (Eastern United States, 30-42N, 70-92W), and boreal (Siberia, 60-70N, 80-140E). The accuracy of these spatially restricted models was generally higher than the reference model (MODEL3) even though the numbers of training images was almost the same. Globally, there are several different climatic zones with different interannual trends. Our results may suggest that the DNN is able to capture climatic trends more successfully when the target area is restricted to a single climatic zone. Among climatic regions, Amazon (tropical rain forests) shows particularly high accuracy, whereas Siberia (boreal forests) has relatively low accuracy. This may be caused by the heterogeneity within the climatic regions. When interannual trends in temperature are homogeneous within the climatic region, we expect higher accuracy. This is understandable from the viewpoints of artificial intelligence and computer vision. For example, to construct a model of human faces, numerous training images are required because human faces are heterogeneous. On the other hand, a homogeneous object such as the trade mark of Coca Cola can be identified relatively easily with a few training images with sample size amplification by modifying colors and shapes.

In experiments with temporal restrictions (MODEL18–MODEL24), the accuracy of the models was in the range 95.0–96.8 for MODEL18 (1900s) to MODEL22 (1940s); however, for MODEL23 (1950s) to MODEL24 (1960s), the accuracy was above 98%. This may be because in MODEL23 and MODEL24, more than 90% of the training images were classified as RISE because global warming became obvious in the latter half of the 20th century. This bias may make predictions easier. Another reason is the data quality; we assume that the quality of the data is improving gradually in the temporal range and this makes predictions more reliable. Although MODEL18 (1900s) also has a bias toward RISE, the accuracy is not particularly high possibly because of the low data quality in the early 20th century.

## Discussion

In this study, we showed that, without physics-based mechanisms, a top-down forecast model can successfully predict rise or fall in temperature in a decadal timescale. Although this study has limitations in predictability because only two classes (RISE or FALL) are used for forecast, this top-down approach can be a meaningful measure for climate change studies. We suggest that this top-down approach should be used together with physics-based bottom-up approaches because these approaches can work in a complementary manner. This study is different from the preceding top-down approach using probabilistic forecast (Sévellec and Drijfhout, 2018) in that we did not need to make any assumptions related to climatology; we simply prepared two-dimensional figures using climate data and fed them to a DNN.

**Figure 4:**
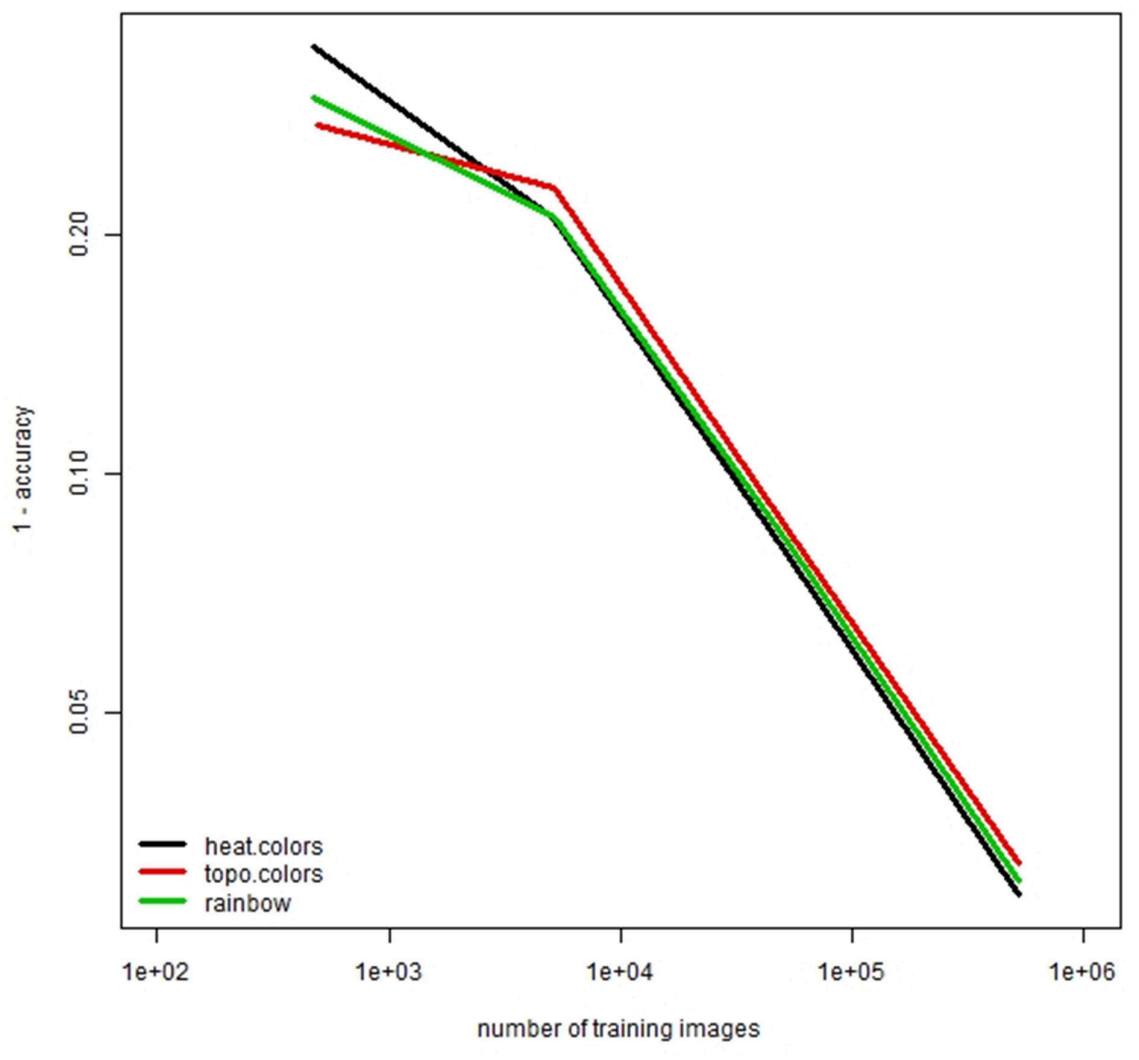
Comparison of accuracy for different colors. Note that both horizontal and vertical axes are logarithmic axes.

Different color schemes lead to some differences in the model performance (Figure 4). We noticed that the rainbow color scheme (see Figures 2I–L) gives more information to human observers but this may not be the case for an artificial intelligence system constructed using a DNN model. When number of training images is small, the performance of the default color scheme (heat.colors) is not good. With large numbers of training images, the performance of heat.colors was better than the other color schemes. These findings may be insightful for various studies on artificial intelligence.

This study can be enhanced with several modifications. For example, we employed LeNet among choices of CNN because of its simplicity. We did not change the default parameter settings of NVIDIA DIGITS 6.0 to show the robustness of our concept. However, other networks such as AlexNet (Krizhevsky et al., 2012) or GoogLeNet (Szegedy et al., 2014) with systematic parameter tuning and multifold cross validation can be used for further improvements in forecasting. Moreover, although this study is a two-class classification in temperature trend (RISE or FALL), it can be possible to use ca. 5 temperature classes for better forecasting. There are advantages and disadvantages for this study. Since this is a top-down approach, it is difficult to measure whether our approach would perform well under novel conditions. This limitation is universal for phenomenological forecasting. Therefore, we suggest that climatic forecasting should integrate both the top-down approach such as this study and the bottom-up approach based on physics.

## Conclusion

This study uncovers several insightful research topics. For example, the models constructed in this study can be used to forecast future trends in temperature. Moreover, along with the mean monthly temperature, other meteorological datasets such as maximum and minimum temperatures and precipitation can be used to generate images for DNN training. This may further improve the model performance. We also suggest using temperature data from simulation studies such as ESM. By doing this, we may be able to show whether climate simulations generate interannual trends similar to observed data. Overall, because climate change is a very important problem, many different approaches should be used to address it. We hope that our DNN-based, top-down approach can be one such novel approach.

## Author Contributions

TI contributed image processing, coding DNN, and study design. YO contributed executing experiments.

### Conflict of Interest Statement

The authors declare that the research was conducted in the absence of any commercial or financial relationships that could be construed as a potential conflict of interest.
